# Alteration of Endothelins: A Common Pathogenetic Mechanism in Chronic Diabetic Complications

**DOI:** 10.1080/15604280214939

**Published:** 2002

**Authors:** Subrata Chakrabarti, Zia Ali Khan, Mark Cukiernik, Gen Fukuda, Shali Chen, Suranjana Mukherjee

**Affiliations:** 1 Department of Pathology The University of Western Ontario Ontario London Canada; 2 Department of Pathology The University of Western Ontario Dental Sciences Building Room 4011, ON London N6A 5C1 Canada

## Abstract

Endothelin (ET) peptides perform several physiological, vascular, and nonvascular functions and are widely distributed in a number of tissues. They are altered in several disease processes including diabetes. Alteration of ETs have been demonstrated in organs of chronic diabetic complications in both experimental and clinical studies. The majority of the effects of ET alteration in diabetes are due to altered vascular function. Furthermore, ET antagonists have been shown to prevent structural and functional changes induced by diabetes in animal models. This review discusses the contribution of ETs in the pathogenesis and the potential role of ET antagonism in the treatment of chronic diabetic complications.


*Int. Jnl. Experimental Diab. Res.*, 3:217–231, 2002
Copyright © 2002 Taylor & Francis
1560-4284/02 $12.00 + .00
DOI: 10.1080/15604280290013982

Taylor & Francis healthsciences

### Alteration of Endothelins: A Common Pathogenetic Mechanism in Chronic Diabetic Complications

**Subrata Chakrabarti, Zia Ali Khan, Mark Cukiernik, Gen Fukuda, Shali Chen, and Suranjana Mukherjee**

*Department of Pathology, The University of Western Ontario, London, Ontario, Canada*

**Endothelin (ET) peptides perform several physiological, vascular, and nonvascular functions and are widely distributed in a number of tissues. They are altered in several disease processes including diabetes. Alteration of ETs have been demonstrated in organs of chronic diabetic complications in both experimental and clinical studies. The majority of the effects of ET alteration in diabetes are due to altered vascular function. Furthermore, ET antagonists have been shown to prevent structural and functional changes induced by diabetes in animal models. This review discusses the contribution of ETs in the pathogenesis and the potential role of ET antagonism in the treatment of chronic diabetic complications.**

**Keywords** Atherosclerosis; Cardiomyopathy; Diabetic Complications; Endothelins; Nephropathy; Neuropathy; Retinopathy

Globally, diabetes is considered as a major threat to human health in the 21st century [1]. It is estimated that about 6% of the North American population suffers from diabetes mellitus [2, 3]. Chronic diabetic complications constitute a group of diseases responsible for substantial morbidity and mortality, and prevention of such complications is a key issue in the management of the diabetes epidemic [1–4]. Therapeutic modalities for diabetes have evolved a great deal. However, most people with this disorder go on to develop complications leading to damage to various body tissues. These complications include diabetic retinopathy, nephropathy, neuropathy, cardiomyopathy, and macroangiopathic complications such as atherosclerosis. The macrovascular complications are not diabetes specific but are more pronounced in diabetes. Diabetic complications arise primarily because of hyperglycemia-induced metabolic changes leading to changes in the structural and functional properties of macromolecules [5, 6]. Recent advances have identified secondary factors that play key roles in the development and progression of these complications. Some of the factors that participate in the pathogenesis of diabetic complications include protein kinase C (PKC) activation, nonenzymatic glycation, oxidative stress, and alterations in growth factor and vasoactive factor expression. Several of these factors may subsequently lead to further endothelin (ET) activation in diabetic subjects.

### ETs AND THEIR RECEPTORS

ETs are potent vasoactive factors. These 21–amino acid peptides cause vasodilation at lower concentrations and sustained contraction at high concentrations. Three isoforms of ETs exist: ET-1, ET-2, and ET-3 [7]. These peptides are encoded by 3 distinct genes. The encoded precursor proteins are spliced by endopeptidases to produce big ETs. A group of proteins called endothelin-converting enzymes (ECEs) then convert these big ETs into mature ET peptides. Two ECEs have been cloned, ECE1 and ECE2 [8, 9]. ECE1 has 4 isoforms (ECE1a–d) and is mainly located on the cell surface, with the exception of ECE1b [10–13]. ECE1b and ECE2 have been localized intracellularly in close proximity to the Golgi network [12].

ETs act on specific receptors ($ET_A$ and $ET_B$). $ET_A$ has high affinity for ET-1 and ET-2 but low affinity for ET-3 and is primarily involved in vasoconstriction [14]. $ET_B$, on the other hand, is equally responsive to all isoforms and is involved in vasodilation by increasing nitric oxide (NO) generation by endothelial cells

Received 20 January 2002; accepted 4 June 2002.
These studies were supported by grants from Canadian Diabetes Association in honor of Margaret Francis and by Canadian Institute of Health Research.
Address correspondence to Dr. Subrata Chakrabarti, Department of Pathology, University of Western Ontario, Dental Sciences Building, Room 4011, London, ON, Canada N6A 5C1. E-mail: schakrab@uwo.ca

[15]. In some cells, ET receptors are coupled to voltage-gated calcium channels, while in others to the activation of phospholipase C. The vasoconstrictive property is due to calcium increase in smooth muscle cells, which results from activation of phospholipase C and production of diacylglycerol and inositol trisphosphate. The differential responsiveness of $ET_A$ receptors to various agonists and antagonists suggests that subtypes of the receptor exist [16–24]. However, no conclusive study has favored the postulate. Radioligand binding studies conducted in the late 1990s have contradicted the existence of $ET_A$ subtypes [25, 26]. This suggests that $ET_A$ responsiveness to various stimulators and inhibitors depends on the intrinsic properties of the ligands. However, similar studies on potencies of ET antagonists on $ET_B$ receptor have suggested the existence of 2 receptor subtypes, $ET_{B1}$ and $ET_{B2}$ [23, 24, 27, 28].

Hypoxia and ischemia are 2 important conditions that may lead to ET up-regulation [29, 30]. However, at the molecular level, several important intracellular molecules may regulate ET mRNA expression in health and disease [31–38]. Majority of the ET-related effects in the target organs of diabetic complications may be mediated via its effects on the vasculature. Nonvascular effects of ETs in the pathogenesis of diabetic complications, although potentially of importance, have not been studied in detail. A diagrammatic representation of the regulation of ET-1 mRNA expression and the mechanisms of ET actions on endothelial and smooth muscle cells are outlined in Figure 1.

### MECHANISMS OF ET ALTERATION IN DIABETES

Several biochemical abnormalities secondary to hyperglycemia may lead to ET alteration in the target organs of diabetic complications. The metabolic pathways leading to ET activation include PKC activation, altered redox state, as well as

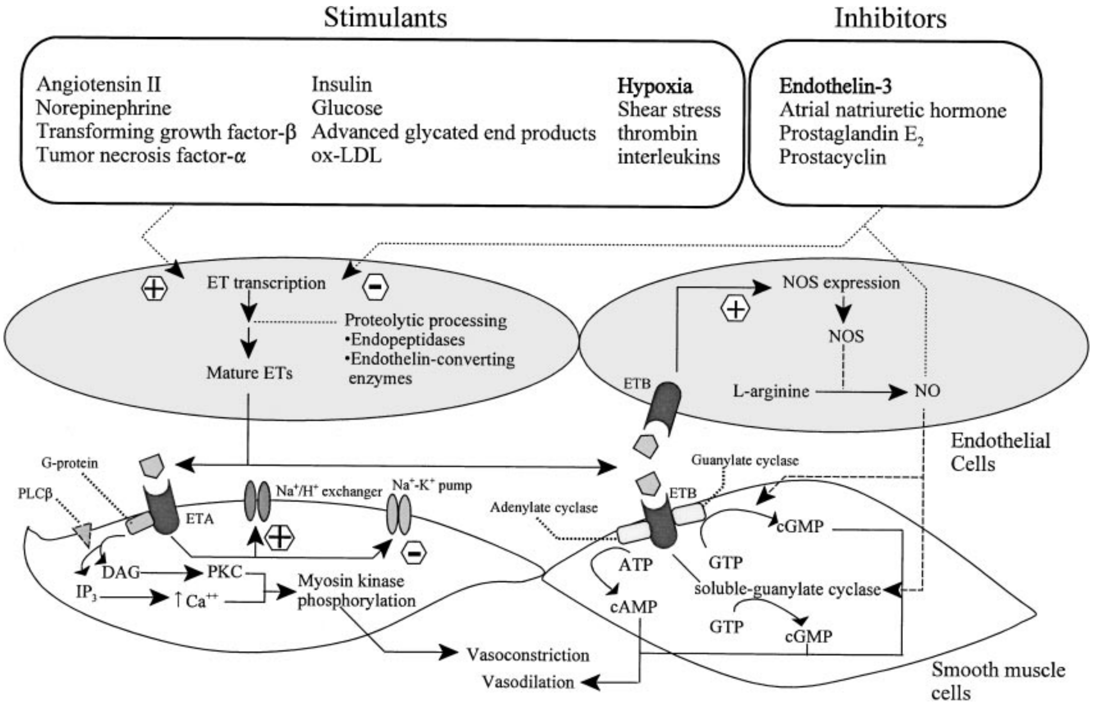

**FIGURE 1**

Vasoactivity of endothelins (ETs). Various stimulators and inhibitors regulate transcription of ETs. The precursor peptides are acted upon by endopeptidases and ET-converting enzymes to produce mature ET peptides. These ET peptides act on G-protein–coupled receptors on endothelial and smooth muscle cells. Interaction of ET with $ET_B$ receptor on endothelial cells leads to increased nitric oxide synthase expression and nitric oxide production. Nitric oxide has an inhibitory effect on ET transcription. In addition, nitric oxide can increase soluble guanylate cyclase activity in smooth muscle cells. Activated guanylate cyclase leads to increases in cGMP and a reduction in intracellular $Ca^{++}$, resulting in vasodilation. ETs can also activate phospholipase C (PLC) in smooth muscle cells via G-protein–coupled $ET_A$ or $ET_B$ receptors. Activation of PLC increases hydrolysis of phosphatidylinositol-4,5-bisphosphate ($PIP_2$) to release 2 second messengers, 1,2-diacylglycerol (DAG) and 1,4,5-inositol trisphosphate ($IP_3$). DAG activates PKC and $IP_3$ increases intracellular calcium. Elevated intracellular calcium leads to myosin kinase phosphorylation and results in contraction. ETs can also regulate the activity of $Na^+/H^+$ exchanger and $Na^+,K^+$-ATPase on smooth muscle cell surface.

alteration of other vasoactive factors. We will briefly discuss the mechanisms of these factors leading to ET alteration in diabetes.

### PKC Activation

PKC activation assumes a central role in hyperglycemia-induced vascular disorders. High glucose concentrations can induce the production of diacylglycerol and activation of PKC [39–41]. PKC activation has been implicated in hyperglycemia-induced vascular permeability and flow changes, expansion of extracellular matrix, and in the production of various growth factors and cytokines [42–48]. One isoform in particular, PKC$\beta$, has been shown to be activated in all tissues affected by chronic diabetes. PKC activation also leads to activation of phospholipase A and production of arachidonic acid metabolites. Furthermore, PKC activation can result in the impairment of $Na^+$,$K^+$-ATPase and endothelial damage [49, 50]. Studies on PKC activation and ETs have suggested an interaction between the 2 factors. We and others have demonstrated that endothelial cells exposed to ET-1 or phorbol 12-myristate 13-acetate (PMA) (a potent PKC activator) show similar permeability changes as seen in hyperglycemia [42]. These changes are prevented when cells are treated with ET receptor antagonists or PKC inhibitors. Furthermore, structural changes, such as F-actin microfilament assembly secondary to glucose, ET-1, or PKC activation, are also ameliorated by respective inhibition [42].

### Polyol Pathway and Redox State

Early experiments aimed toward elucidating the mechanistic basis of chronic diabetic complications focused on sorbitol accumulation and accompanying cellular damage. Glucose is converted to sorbitol via augmented polyol pathway. The enzyme responsible, aldose reductase, has been shown to be up-regulated in all tissues affected by chronic diabetic complications [43, 51]. Increased intracellular concentrations of sorbitol can cause osmotic changes, cell swelling, and abnormalities in myoinositol metabolism and can lead to impairment of $Na^+$,$K^+$-ATPase. Aldose reductase inhibition has been shown to prevent hyperglycemia-induced damage in diabetic retinopathy, neuropathy, and nephropathy to some extent [52, 53]. The mechanism by which aldose reductase inhibition prevents development of vascular complications is not fully understood. Aldose reductase requires NADPH for the conversion of glucose to sorbitol. Sorbitol is then converted to fructose by sorbitol dehydrogenase. The latter step requires $NAD^+$ reduction for the enzymatic conversion. This suggests that an imbalance in the redox state, that is, altered NADH:NAD and NADPH:NADP might cause endothelial dysfunction secondary to increased aldose reductase activity [43, 51, 54]. Interestingly, depleted NADPH may also lead to reduced NO production as the enzymatic reaction of NO synthesis requires NADPH, which may result in ET up-regulation.

### Nonenzymatic Glycation

Nonenzymatic glycation and generation of advanced glycated end (AGE) products has been demonstrated as an important factor in the pathogenesis of chronic diabetic complications [43, 55–57]. Glucose, fructose, and the product of the pentose phosphate pathway may participate in nonenzymatic glycation [56–58]. AGEs and the reactive intermediates may have widespread biological actions [55–59]. AGEs may further increase oxidative stress and endothelial damage [55–59]. Exogenous administration of superoxide dismutase has been shown to reduce hyperglycemia-induced endothelial permeability and accompanying vascular dysfunction [59–62]. In addition, AGEs can form cross-links with collagen in the extracellular matrix, reduce arterial compliance, and alter gene expression of several important intracellular molecules [55–57]. Aminoguanidine, a specific inhibitor of nonenzymatic glycation, has been shown to inhibit the development of retinopathy in diabetic dogs [63–65].

Several receptors for AGE have been identified. Both AGEs and their receptors have been localized to the target organs of diabetic complications [55–57]. These receptors are found on many cells, including endothelial and smooth muscle cells. Interaction of AGEs with these receptors leads to mitogen activated protein kinase (MAPK) kinase signaling and nuclear factor kappa B (NF-$\kappa$B) activation. AGE-mediated NF-$\kappa$B activation has been shown to increase ET-1 expression [66]. Activation of NF-$\kappa$B secondary to nonenzymatic glycation has also been linked to reduced NO, which would positively affect ET expression [67].

### Nitric Oxide and Oxidative Stress

NO is a potent vasodilator formed from L-arginine by NO synthase (NOS) [68]. This enzyme has 2 major isoforms, calcium-calmodulin–dependent endothelial NOS (also called eNOS) and calcium-calmodulin–independent inducible NOS (also called iNOS). eNOS is expressed constitutively in endothelial cells and some other cells, whereas iNOS is only expressed when cells are stimulated by various factors [69–71]. NO released from endothelial cells acts on smooth muscle cells to increase intracellular cGMP and cAMP. The result of this increase in cGMP and cAMP is decreased calcium, probably via efflux, and dephosphorylation of myosin light chains [72]. Endothelial dysfunction is characterized by the imbalance between contracting and relaxing factors. The mechanism of glucose-mediated endothelial dysfunction is not fully understood, but evidence indicates increased ET production and impaired NO may contribute significantly in this abnormality [73–77]. There is a negative interaction between NO and ET, thereby a reduction in NO leads to increased ET expression.

Generation of free radicals due to glucose autoxidation may also damage proteins [78]. Various lipoxygenase enzymes activated in hyperglycemia may interact with NO, forming peroxynitrate and hydroxyl radicals [79, 80]. Augmented mitochondrial production of superoxide ions secondary to hyperglycemia has recently been proposed as a unifying initiating mechanism [81, 82]. Increased superoxide ions may inhibit the glycolytic enzyme glyceraldehyde-3-phosphate dehydrogenase (GAPDH), hence excess glucose is deviated to avenues of metabolism, leading to the activation of PKC, augmented polyol pathway, generation of methyl glyoxal, and AGE formation, as well as increased flux through hexosamine pathway [81, 82]. In several cell types, overexpression of superoxide dismutase (SOD) has been shown to prevent specific glucose-induced abnormalities [81, 83, 84]. Furthermore, in transgenic mice overexpressing MnSOD, diabetes-induced lesions were prevented [81].

### Other Factors

Increased vascular permeability is one of the characteristic features of endothelial dysfunction. This permeability alteration is due to the expression of vascular endothelial growth factor (VEGF), which has been demonstrated in diabetes [85–90]. VEGF is a member of a large family of proteins, with 5 isoforms generated by alternative splicing [91, 92]. The mechanism by which VEGF carries out the permeability and proliferative changes seems to involve PKC [89]. Various vasoactive peptides can also induce VEGF production [47]. VEGF activates 1,4,5-inositol triphosphate ($PI_3$) kinase and phospholipase C gamma (PLC$\gamma$). Activation of PLC$\gamma$ increases 1,2-diacylglycerol (DAG) and activates PKC$\alpha$ and PCK$\beta$. Oral administration of PKC inhibitors, specifically PKC$\beta$, prevents VEGF-mediated retinal permeability and endothelial proliferation [89]. ETs are probably also important in mediating glucose-induced increased permeability [42]. We and others have demonstrated that VEGF may further increase ET expression in endothelial cells of the diabetic rat retina [42].

A large number of studies indicate the role of angiotensin in the development of diabetic micro- and macrovasculopathy. Angiotensin II has mitogenic effects on smooth muscle cells and can lead to increased extracellular matrix (ECM) protein synthesis by these cells [93]. Recent reports indicate that there might be an interaction between the renin-angiotensin pathway and the ET system [94]. In vitro studies have demonstrated an angiotensin II–mediated increase in ET expression [95].

### PATHOPHYSIOLOGICAL ROLE OF ETs IN DIABETES

ET alteration, in diabetes, may lead to several functional and structural effects that are important in the development of several chronic complications. Both glucose and insulin, potentially of importance in insulin resistance, are ET-1 up-regulators [95, 96]. We and others have demonstrated that in endothelial cells and in several target organs of diabetic complications, ETs are up-regulated [97–104]. Studies in human diabetes with respect to serum ET levels have shown a widespread variation, ranging from increased to unchanged to decreased [105–114]. Several factors may be responsible for such discrepancy. However, as ETs act as autocrine and paracrine factors, plasma levels may not be a good indicator of their biological activity [115, 116]. Increased ETs may lead to increased vascular permeability and altered of blood flow [42]. In addition, increased ET levels may contribute greatly to hypertension in diabetics. Studies conducted to determine the role of ETs in essential hypertension have shown inconsistent data. However, there does seem to be a positive association between the 2 factors [117]. ETs may also lead to increased ECM protein production in diabetes via activation of transcription factors NF-$\kappa$B and activated protein (AP-1) [118, 119]. Furthermore, they may potentially play important roles in angiogenesis [120–122]. Some of the major effects of ET alteration in diabetes are outlined in Figure 2. We will discuss the role of ETs in specific diabetic complications below.

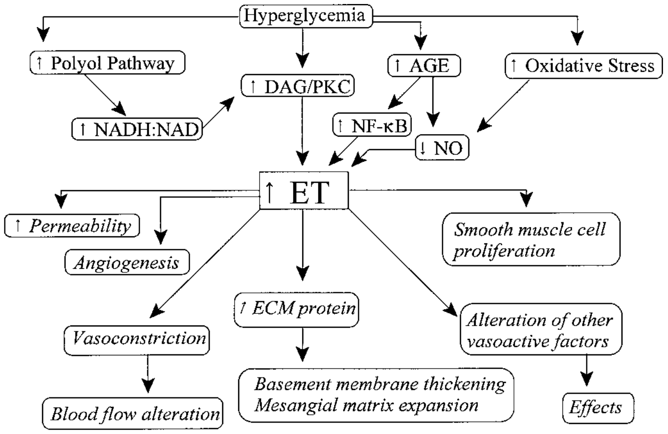

**FIGURE 2**
Mechanism and consequences of ET alteration in diabetes. A schematic outline of various hyperglycemia-induced pathways leading to increases in ET levels (upper half of figure). Some of the major effects of increased ET levels are also presented (shown in italics; lower half of the figure).

### DIABETIC RETINOPATHY

Diabetes is the most important systemic disease causing blindness [123, 124]. Diabetic retinopathy is classified in various progressive stages, namely, nonproliferative (background) retinopathy, preproliferative (severe or advanced background) retinopathy, and proliferative retinopathy.

The retina is comprised of several tissue types, including neural tissue with respective support cells and vascular tissue.

Diabetic retinopathy predominantly affects the vascular components of the retina. Pathological changes in background diabetic retinopathy include capillary basement membrane thickening, pericyte loss, microaneurysms, acellular capillaries, increased capillary permeability with exudate deposits, and retinal microinfarcts. In advanced proliferative retinopathy, neovascularization develops with its devastating consequences. ETs have demonstrated an effect on both vascular and neural tissue components in the retina [115, 116]. Due to the multifactorial nature of the pathogenesis of diabetic retinopathy, ETs can be up-regulated and further interact with a variety of other factors to produce pathological changes [125, 126].

Although ETs are believed to work in an autocrine and paracrine manner, human type 1 diabetes has demonstrated both decreased [127, 128] and increased [108, 114, 129] plasma ET-1 levels. Patients with type 2 diabetes have demonstrated increased [109, 111, 130] and unchanged [131, 132] plasma levels. Positive correlations between increased ET-1 levels and microangiopathy have been demonstrated [109, 110, 133–135]. Furthermore, plasma ET-1 levels can be returned to normal levels in the diabetic rats by restoring metabolic control [98].

The 2 ET isoforms expressed by the retina are ET-1 and ET-3. In the retina, ETs have been demonstrated to localize in the optic nerve, vascular and extravascular sites of the retina, and the uveal tract [101, 102, 135]. Increased mRNA and protein expression of the 2 proteins have been produced in animal diabetes [102, 136, 137]. Expression of the receptor isoforms, $ET_A$ and $ET_B$, have demonstrated mixed results, depending upon the tissue preparations. If the neural retina is isolated alone, the levels appear to decrease and then increase [137]. If both neural and vascular components are analyzed, then receptor levels appear to increase [101, 136–138]. ETs can modulate the vascular tone of blood vessels, depending upon the concentration. At low concentrations, ETs cause vasodilation, and at high concentrations, vasoconstriction [139–143]. The vasodilation is mediated by the binding of ET-1 and ET-3 to the $ET_B$ receptor. Subsequently, through a G-protein–mediated pathway, NO or prostacyclins are released by endothelial cells [15, 144–147]. The vasoconstriction is mediated through ET-1 binding with $ET_A$ receptors located on smooth muscle cells [14, 15, 147, 148]. Our own research has demonstrated a significant up-regulation of ET-1 and ET-3 in conjunction with increased resistivity index, in the retina of 4-week streptozotocin (STZ)-induced diabetic animals [101]. The resistivity index is an indirect measurement of retinal vasoconstriction. In short- term diabetes, increased resistivity index was corrected by treatment with a dual ET receptor blocker, bosentan. However, in longer term diabetes, such vasoconstriction was not present [101].

ETs possess mitogenic properties and may regulate ECM protein synthesis [116]. Our research has illustrated that ET receptor blockade with bosentan prevents the diabetes-induced up-regulation of basement membrane proteins, fibronectin, and collagen alpha-1 (IV) mRNA, as well as increased capillary basement membrane thickening in both glycemic and galactosemic animals [119]. Recently, we have further demonstrated that diabetes-induced increased ECM protein production works through NF-$\kappa$B and AP-1 activation [118]. NF-$\kappa$B activation could also act as a mechanism of ET-1 up-regulation [66].

Along with the direct actions, ETs show widespread interactions with other vasoactive peptides, modulating their effects and vice versa. ET mRNA levels can be up-regulated through the actions of thrombin, transforming growth factor-$\beta$ (TGF-$\beta$), interleukin-1, epinephrine, angiotensin II, metallothionein, and vasopressin [31, 36, 149–151]. Furthermore, we have demonstrated a costimulatory interaction between VEGF and ETs in cultured endothelial cells, as well as ET-1 up-regulation by NO inhibition [42]. Interestingly, in the retinas of short-term diabetic animals, increases in resistivity index can be corrected with the use of an ET blocker [101], a VEGF signal antagonist [152], or a $Na^+/H^+$ exchanger-1 (NHE-1) antagonist [91]. Although VEGF antagonism increased iNOS mRNA expression, NHE-1 blockade reduced ET-1 mRNA expression in these animals [152, 153], suggesting that several interactive molecules may indeed be responsible for the control of vascular tone in the retina.

The present data would suggest that ETs may play an important role in the pathogenesis of diabetic retinopathy by altering blood flow, increasing vascular permeability, increasing ECM protein synthesis, and basement membrane thickening via the mechanisms stated above. ETs may work in conjunction with other factors in mediating these effects. Recently, ETs have been implicated in the pathogenesis of proliferative diabetic retinopathy [154]. However, such role for ETs needs further investigation.

### NEPHROPATHY

Diabetic nephropathy affects approximately 30% of type 1 diabetic patients [155, 156]. Diabetes remains the most important cause of renal failure in industrialized countries [157]. Glomerular hyperfiltration leading to microalbuminuria is the earliest clinical marker of this disease. With progression of renal damage, patients develop macroalbuminuria and reduced glomerular filtration rate [155–157].

Pathological features of diabetic nephropathy include mesangial matrix expansion, thickening of glomerular capillary basement membrane, and tubulointerstitial fibrosis. In earlier stages, however, there is renal enlargement due to cellular hypertrophy affecting both the glomeruli and tubules. Eventually, the glomerular filtration rates continue to decline and the patients develop arteriolosclerosis and glomerulosclerosis with obliteration of the filtration area due to increased production and decreased degradation of ECM proteins. In the later stages, patients develop characteristic nodular accumulation of extracellular matrix proteins, that is, Kimmelstiel–Wilson nodules [157–161]. Clinically, overt nephropathy manifests as proteinuria in the nephrotic range, hypertension, and other features of renal failure. It has been demonstrated that, similar to other chronic complications, a high blood glucose level is the initiating factor leading to the development of renal damage in diabetes [162, 163]. Furthermore, it has been demonstrated that good glucose control may even reverse the structural changes in the kidneys [162]. Another risk factor in the progression of nephropathy is coexisting systemic hypertension, which leads to high intraglomerular hypertension and hyperfiltration, promoting further tissue destruction and sclerosis [163].

In the kidney, both ET-1 and ET-3 show widespread tissue distribution. ETs are expressed in the endothelium, epithelium, mesangium, the glomeruli, the tubular epithelium, and the collecting ducts and vasa recta [164, 165]. ET binding sites have been localized in these cells and in the interstitial cells. From a physiological perspective, ETs have roles in the regulation of renal blood flow, glomerular filtration, and sodium and water reabsorption [116, 164].

In the kidney of diabetic rats, increased ET-1 mRNA and increased renal ET-1 clearance in association with proteinuria has been demonstrated [166]. Increased $ET_A$ receptors are present in the kidneys of diabetic rabbits [167]. Recent data from our laboratory has demonstrated that 1 month of diabetes causes a significant increase in the mRNA expression of ET-1, ET-3, $ET_A$, $ET_B$, which remained elevated at 6 months of follow-up. Galactose feeding, however, resulted in increased $ET_A$ and $ET_B$ receptor mRNA expression at 1 month, which was accompanied by ET-1 and ET-3 mRNA up-regulation at 6 months. These results suggest differential activation of the components of ET system in diabetes and following galactose feeding. Nevertheless, in both animal models, glucose-induced, ET-mediated microalbuminuria was prevented by treatment with bosentan [165].

The long-term consequences of ET peptides may involve cellular changes requiring differential gene expression and may contribute to long-term nuclear signaling [164]. Previous studies have implicated ETs in having a regulatory link with the components of the ECM, because rat mesangial cells showed that ET-1 can increase the production of ECM components such as laminin and collagen a1 (IV) [168]. Furthermore, diabetes-induced increased expression of glomerular a1(I), a1(III), a1(IV) collagen, laminin B1 and B2, tumor necrosis factor-$\alpha$, platelet-derived growth factor, TGF-$\beta$, and basic fibroblast growth factor can be completely blocked by treatment with an $ET_A$ receptor antagonist [169]. We have recently demonstrated that diabetes and galactose feeding–induced increases in ECM protein mRNA expression, glomerular basement membrane thickening, and mesangial matrix expansion are prevented by bosentan treatment [165]. We have further demonstrated that glucose-induced, ET-mediated increased ECM protein fibronectin synthesis is mediated via transcription factors NF-$\kappa$B and AP-1 [118].

Diabetic nephropathy seems to occur as a result of interactive metabolic factors secondary to hyperglycemia. Several biochemical abnormalities acting secondary to hyperhexosemia may lead to augmented ET expression in the kidneys. ETs, by virtue of the modulation of blood flow as well as ECM protein production, are of importance in diabetic nephropathy. PKC activation secondary to hyperglycemia may be one of the factors up-regulating ET-1 mRNA expression. ET-1, on the other hand, is a PKC stimulator [116, 136]. TGF-$\beta$ in recent years has been established as one major factor producing renal lesions in diabetes [161]. PKC-dependent factors, such as TGF-$\beta$, can act as an ET-1 stimulator and may play a significant role in ET alteration [116, 164]. It has been demonstrated that PKC$\beta$ inhibition results in the prevention of ECM protein synthesis and TGF-$\beta$ production in the kidney [170]. We have previously demonstrated that general PKC inhibition or PKC$\beta$ inhibition prevents the high glucose–induced increased permeability and ET-1 expression in endothelial cells [42]. ET-mediated increased ECM protein production may represent one of the long-term effects of ET-1, possibly mediated via several kinases, including PKC, MAP kinase, and serine/threonine kinase, and transcription factors, which warrant further investigations [115].

### NEUROPATHY

Both the somatic and autonomic nervous system can be affected by diabetes, causing a variety of symptoms. Diabetes is a major cause of peripheral neuropathy in the western world [171]. A significant number (60% to 70%) of diabetic patients show variable degrees of nerve damage. At the severe end of the spectrum, diabetic nerve disease is a major cause of lower extremity amputation [162]. Broadly, diabetic neuropathy can be classified as mononeuropathies or polyneuropathies. The mononeuropathies may affect peripheral or cranial nerves and can involve single nerves or may affect multiple nerves (mononeuritis multiplex) [171, 172]. Sensory, motor, or autonomic nervous systems may be affected by polyneuropathies. Chronic sensorimotor polyneuropathy is the most common type of neuropathy. It is manifested as progressive gloves and stocking anesthesia, paresthesia, or hyperasthesia, impaired balance, proprioception, and vibration. Although motor weakness is not pronounced, wasting of small muscle and loss of reflex activity are manifested. Foot ulceration and other neuropathic changes may subsequently develop. Impaired nerve conduction velocity is a key electrophysiological feature of diabetic neuropathy. Autonomic neuropathy may produce gastrointestinal or urological motility problems and postural hypotension [171, 172]. Acute sensory neuropathy, although a painful condition, usually leads to complete recovery. Proximal motor neuropathies, also known as amyotrophy, are manifested as acute onset of pain and weakness of proximal muscles.

Impaired activity of PKC and $Na^+$,$K^+$-ATPase as a result of reduced phosphoinositide metabolism in the peripheral nerve has been demonstrated in diabetes [173]. This is in sharp contrast to the retinal findings where activation of PKC has been established [43, 44]. These data indicate that pathogenetic pathways may be influenced by the tissue microenvironment in hyperglycemia and may vary in target organs of diabetic complications. Although peripheral nerves from diabetic animals show reduced DAG levels [173–175], PKC inhibitors prevent diabetes-induced reduced neuronal $Na^+$,$K^+$-ATPase activity [176]. It is possible that impaired phosphoinositide metabolism, in the peripheral nerves in diabetes, may influence ET-mediated signal transduction [177]. In addition, reduced NO production, as demonstrated in the vasculature of the peripheral nerve in diabetes, may also lead to increased ET synthesis [178]. However, these notions need confirmation by specific experiments. Reduced endoneurial blood flow and nerve conduction velocity deficit in the STZ-induced diabetic rats were prevented by a specific $ET_A$ antagonist and by blockade of both $ET_A$ and $ET_B$ receptors [104, 179]. We have demonstrated that immunoreactivity of ET-1 and ET-3 is increased in the peripheral nerve in diabetes [180]. The predominant effect of ET-1 in neuropathy may be mediated via the vascular consequences. Recently, it has further been shown that susceptibility to ET-induced multifocal ischemic damage of the peripheral nerves in diabetic animals is much greater compared to nondiabetic animals [181]. No data, however, are yet available that demonstrate the effects of ET blockade on later changes in diabetic neuropathy such as nerve fiber loss.

### MACROANGIOPATHY

Among the diabetic complications, macroangiopathy is the major cause of mortality in diabetic patients. Type 2 diabetes mellitus is one independent risk factor for atherosclerosis along with hyperlipidemia, hypertension, and smoking. Coronary atherosclerosis leading to myocardial infarction is a leading cause of death in the diabetic population [182]. In type 1 and type 2 diabetic patients, carotid stenosis secondary to an increase intimal and medial wall thickness has been associated with an increased risk of stroke. Furthermore, a direct relationship between carotid artery wall thickness and blood glucose levels in diabetic patients has been noted [183]. Lower extremity arterial disease (LEAD) has been considered to be among the major indications for amputation in individuals with type 1 and type 2 diabetes. The progression of LEAD in diabetes is compounded by such comorbidity as peripheral neuropathy and insensitivity of the feet and lower extremities to pain and trauma [184].

Alterations of the plasma ET-1 levels have been demonstrated in several diseases associated with endothelial dysfunction, that is, diabetes, hypertension, and atherosclerosis [185]. ET-1 is released by endothelial cells and causes phenotypic modulation of the vascular smooth muscle cells from contractile type to synthetic type [186]. ET-1 also causes intimal vasodilation by activation of $ET_B$ receptors on endothelial cells [187]. In addition to its vasoconstrictor properties, ET-1 is a potent mitogen and induces vascular smooth muscle cell proliferation and medial thickening. The importance of ET-1 in diabetes-associated vascular hypertrophy was initially suggested by studies reporting an increased release of this peptide from mesenteric vessels in diabetic rats [188], as well as in type 2 diabetic patients with atherosclerotic macro- and microvascular diseases [189]. Recent studies have demonstrated increased ET-1 expression in the endothelium, adventitia, and the media of diabetic mesenteric vessels in rats [190] and in type 2 diabetic patients with macroangiopathy [191]. High levels of plasma ET-1 were also demonstrated in type 2 diabetic patients with atherosclerosis [134].

Although the expression of prepro ET-1 mRNA was significantly enhanced in aortas from STZ-induced diabetic rats, some investigators demonstrated that the contractile response of the aorta to ET-1 was weaker in STZ-induced diabetic rats than in nondiabetic controls, in association with a down-regulation of ET receptors. Impaired ET-induced signal transduction mechanisms are suggested to be present in these animals [192]. In addition to the direct effect of ETs, there are several interactions between various neurohormonal pathways, including the renin-angiotensin system and ET. In a nondiabetic model, it has been demonstrated that angiotensin II–induced vascular hypertrophy can be attenuated by ET receptor antagonism [193, 194]. ETs may also have roles in mediating increased noradrenaline-mediated vasoconstriction in the aorta and mesenteric vessels of diabetic rats [94]. Furthermore, hyperinsulinemia in the context of insulin resistance may further augment ET-1 action, as insulin is a stimulator of ET-1 production, leading to further smooth muscle proliferation [116, 164]. The present data suggest that ETs may play an important role in the pathogenesis of diabetic macroangiopathy by influencing smooth muscle cell phenotype and functional properties. Further well-designed experimental and clinical investigations are necessary to establish the exact mechanisms.

### CARDIOMYOPATHY

Cardiac affection in diabetes may occur due to the consequences of coronary artery disease, autonomic neuropathy, and diabetic cardiomyopathy, either alone or in combination. Primary affection of cardiomyocytes in diabetes, that is, diabetic cardiomyopathy, can act as an independent factor affecting the cardiac structure and function and may also modulate prognosis of other complications such as ischemic heart disease [195, 196]. It was demonstrated that diabetic patients had larger mean diameters of ventricular myocardial cells and higher percentage of interstitial fibrosis than control subjects. Morphological changes in diabetic cardiomyopathy include myocyte hypertrophy and/or necrosis, interstitial and perivascular fibrosis, and capillary basement membrane thickening [196, 197]. Functional abnormalities involve both the systolic and diastolic properties of the myocardium, such as impaired relaxation, reduced compliance with elevated end-diastolic pressure, cardiac hypertrophy, and chamber dilatation [197, 198].

ETs play important roles in several cardiovascular diseases, including diabetic cardiomyopathy, by modulating functional properties of cardiomyocytes and the microvasculature [100]. In the heart, both cardiomyocytes and endothelial cells produce ET-1. Cardiac myocytes have high ET-1 binding affinity sites [199–202]. ET-1 produces pronounced positive inotropic and chronotropic effects on the heart [197–204]. A duration-dependent alteration of chronotropic and inotropic responses of ET-1 was demonstrated in the isolated atria of the diabetic rat [205]. Short-term hyperglycemia increased the expression of prepro ET-1 in the heart of STZ-diabetic rats [206–209]. We have demonstrated a significant up-regulation of ET-1 and $ET_A$ and $ET_B$ receptor mRNA expression, as well as increased ET immunoreactivity and ET receptor density in hearts of 6-month diabetic rats [100]. These changes were associated with focal apoptosis of cardiomyocytes, scarring of the myocardium, and increased fibronectin and collagen a1(IV) mRNA expression. Furthermore, such diabetes-induced abnormalities were completely prevented by bosentan [100]. In humans, high plasma levels of ET-1 is thought to play an important role in the pathogenesis of diastolic dysfunction in diabetic patients with cardiac autonomic neuropathy [207] . It has been further demonstrated that reperfusion following cardioplegia during coronary artery bypass grafting procedure can trigger the release of ET-1 in diabetic patients [208], which may further contribute to significant cardiovascular demise in diabetic patients.

Some of the effects of ET-1 on the heart and vasculature could be partially mediated via activation of NHE-1, the major proton pump mechanism in the heart, through the $IP_3$-DAG pathway [209, 210]. We have demonstrated that both ET-1 and NHE-1 play important roles in the pathogenesis of diabetic heart disease. NHE-1 may act as the downstream mediator in the development of ET-mediated functional and structural changes in diabetic myocardium [211]. PKC activation may be one of the pathways leading to ET up-regulation in the heart in diabetes [43, 44, 50]. However, several other factors may also be involved in the up-regulation of ET-1 expression in diabetes. ET-1 interacts with other potent vasoactive substances, such as NO and VEGF [211–214]. Increased VEGF in diabetes may also lead to increased ET-1 expression [42]. On the other hand, nonenzymatic glycation and oxidative stress reduces NO production in diabetes, which in turn increases ET-1 expression [79]. We have recently demonstrated that diabetes-induced reduction in NOS expression and NO activity in the heart may be corrected by treatment with bosentan [215].

### CONCLUSION

Evidence gathered so far indicates that pathogenetic mechanisms leading to chronic diabetic complications are complex. Several factors may be simultaneously activated in response to hyperglycemia, and an intricate interplay occurs among such factors. Furthermore, tissue-specific variations exist due to the influence of the tissue microenvironment. This concept further explains prevention and/or delay of chronic diabetic complications with good blood glucose control and failure of adjuvant treatments that block a single pathway, such as aldose reductase inhibition in clinical trials [216, 217]. ETs, due to their widespread tissue distribution, multiple functional capabilities, and alteration in the target organs of diabetic complications, may play significant roles as effector molecules in chronic diabetic complications. Abnormal metabolic pathways secondary to hyperglycemia, such as PKC activation, nonenzymatic glycation, oxidative damage, as well as augmented polyol pathway may, in part, directly or indirectly contribute to the alteration of ETs. ETs may further affect activity of other vasoactive factors. Evidences gathered from multiple animal experiments in several laboratories, indeed, indicate that ETs are important in the pathogenesis of several chronic diabetic complications. ET antagonism may be a potential therapeutic modality in the treatment of chronic diabetic complications, such as retinopathy, nephropathy, neuropathy, and cardiovascular complications. These data, however, have to be further confirmed by additional long-term studies in experimental animals as well as by thorough well-designed clinical trials.

### REFERENCES

[1] Zimmet, P., Alberti, K. G., and Shaw, J. (2001) Global and societal implications of the diabetes epidemic. *Nature,* **414,** 782–787.
[2] Harris, M. I. (1998) Diabetes in America: Epidemiology and scope of the problem. *Diabetes Care,* **21,** C11–C14.
[3] Harris, M. I., Flegal, K. M., Cowie, C. C, Eberhardt, M. S., Goldstein, D. E., Little, R. R., Wiedmeyer, H. M., and Byrd-Holt, D. D. (1998) Prevalence of diabetes, impaired fasting glucose, and impaired glucose tolerance in US adults: The Third National Health and Nutrition Examination Survey (1988–1994). *Diabetes Care,* **21,** 518–524.

[4] Zimmet, P. (2000). Globalization, coca-colonization and the chronic disease epidemic: Can the Doomsday scenario be averted? *J. Intern. Med.,* **247,** 301–310.

[5] The Diabetes Control and Complications Trial Research Group. (1993) The effect of intensive treatment of diabetes on the development and progression of long-term complications in insulin-dependent diabetes mellitus. *N. Engl. J. Med.*, **29,** 977–986.

[6] Engerman, R., Bloodworth, J. M., Jr., and Nelson, S. (1985) Relationship of microvascular disease in diabetes to metabolic control. *Diabetes,* **26,** 760–769.

[7] Inoue, A., Yanagisawa, M., Kimura, S., Kasuya, Y., Miyauchi, T., Goto, K., and Masaki, T. (1989) The human endothelin family: Three structurally and pharmacologically distinct isopeptides predicted by three separate genes. *Proc. Natl. Acad. Sci. U. S. A.,* **86,** 2863–2867.

[8] Takahashi, M., Matsushita, Y., Iijima, Y., and Tanzawa, K. (1993) Purification and characterization of endothelin-converting enzyme from rat lung. *J. Biol. Chem.,* **268,** 21394–21398.

[9] Emoto, N., and Yanagisawa, M. (1995) Endothelin-converting enzyme-2 is a membrane-bound, phosphoramidon-sensitive metalloprotease with acidic pH optimum. *J. Biol. Chem.,* **270,** 15262–15268.

[10] Shimada, K., Takahashi, M., Ikeda, M., and Tanzawa, K. (1995) Identification and characterization of two isoforms of an endothelin-converting enzyme-1. *FEBS Lett.,* **371,** 140–144.

[11] Valdenaire, O., Rohrbacher, E., and Mattei, M. G. (1995) Organization of the gene encoding the human endothelin-converting enzyme (ECE-1). *J. Biol. Chem.,* **270,** 29794–29798.

[12] Schweizer, A., Valdenaire, O., Nelbock, P., Deuschle, U., Dumas Milne Edwards, J. B., Stumpf, J. G., and Loffler, B. M. (1997) Human endothelin-converting enzyme (ECE-1): Three isoforms with distinct subcellular localizations. *Biochem. J.,* **328,** 871–877.

[13] Valdenaire, O., Lepailleur-Enouf, D., Egidy, G., Thouard, A., Barret, A., Vranckx, R., Tougard, C., and Michel, J. B. (1999) A fourth isoform of endothelin-converting enzyme (ECE-1) is generated from an additional promoter molecular cloning and characterization. *Eur. J. Biochem.,* **264,** 341–349.

[14] Arai, H., Hori, S., Aramori, I., Ohkubo, H., and Nakanishi, S. (1990) Cloning and expression of a cDNA encoding an endothelin receptor. *Nature,* **348,** 730–732.

[15] Sakurai, T., Yanagisawa, M., Takuwa, Y., Miyazaki, H., Kimura, S., Goto, K., and Masaki, T. (1990) Cloning of a cDNA encoding a non-isopeptide-selective subtype of the endothelin receptor. *Nature,* **348,** 732–735.

[16] Bax, W. A., Aghai, Z., van Tricht, C. L., Wasenaar, C., and Saxena, P. R. (1994) Different endothelin receptors involved in endothelin-1- and sarafotoxin S6b-induced contractions of the human isolated coronary artery. *Br. J. Pharmacol.,* **113,** 1471–1479.

[17] Clark, K. L., and Pierre, L. (1995) Characterization of endothelin receptors in rat renal artery in vitro. *Br. J. Pharmacol.,* **114,** 785–790.

[18] Godfraind, T. (1993) Evidence for heterogeneity of endothelin receptor distribution in human coronary artery. *Br. J. Pharmacol.,* **110,** 1201–1205.

[19] Eglazos, A., Cucchi, P., Patacchini, R., Quartara, L., Maggi, C. A., and Mizrahi, J. (1993) Differential effects of BQ-123 against endothelin-1 and endothelin-3 on the rat vas deferens: Evidence of an atypical endothelin receptor. *Br. J. Pharmacol.,* **109,** 736–738.

[20] Ishikawa, H., Haruno, I., Harada, Y., Yoshitomi, T., Ishikawa, S., and Katori, M. (1996) Pharmacological characterization of endothelin receptors in rabbit iris sphincter muscle: Suggestion for the presence of atypical receptors. *Curr. Eye Res.,* **15,** 73–78.

[21] Riezebos, J., Watts, I. S., and Vallance, P. J. (1994) Endothelin receptors mediating functional responses in human small arteries and veins. *Br. J. Pharmacol.,* **111,** 609–615.

[22] Sumner, M. J., Cannon, T. R., Mundin, J. W., White, D. G., and Watts, I. S. (1992) Endothelin $ET_A$ and $ET_B$ receptors mediate vascular smooth muscle contraction. *Br. J. Pharmacol.,* **107,** 858–860.

[23] Warner, T. D., Allcock, G. H., Corder, R., and Vane, J. R. (1993) Use of endothelin antagonists BQ-123 and PD 142893 to reveal three endothelin receptors mediating smooth muscle contraction and the release of EDRF. *Br. J. Pharmacol.,* **110,** 777–782.

[24] Warner, T. D., Allcock, G. H., Mickey, E. J., and Vane, J. R. (1993) Characterization of endothelin receptors mediating the effects of endothelin/sarafotoxin peptides on autonomic neurotransmission in the rat vas deferens and guinea-pig ileum. *Br. J. Pharmacol.,* **110,** 783–789.

[25] Devadason, P. S., and Henry, P. J. (1997) Comparison of the contractile effects and binding kinetics of endothelin-1 and sarafotoxin S6b in rat isolated renal artery. *Br. J. Pharmacol.,* **121,** 253–263.

[26] Nosaka, C., Ishikawa, H., Haruno, I., Yoshitomi, T., Kase, H., Ishikawa, S., and Harada, Y. (1998) Radioligand binding characteristics of the endothelin receptor in the rabbit iris. *Jpn. J. Pharmacol.,* **76,** 289–296.

[27] Douglas, S. A., Beck, G. R. J., Elliot, J. D., and Ohlstein, E. H. (1995) Pharmacologic evidence for the presence of three functional endothelin receptor subtypes in rabbit saphenous vein. *J. Cardiovasc. Pharmacol.,* **26,** S163–S168.

[28] Mizuguchi, T., Nishiyama, M., Moroi, K., Tanaka, H., Saito, T., Masuda, Y., Masaki, T., de Wit, D., Yanagisawa, M., and Kimura, S. (1997) Analysis of two pharmacologically predicted endothelin B receptor subtypes by using the endothelin receptor B knockout mouse. *Br. J. Pharmacol.,* **120,** 1427–1430.

[29] Blauw, G. J., Westendorp, R. G., Srivistava, N., et al. (1995) Hypoxia induced arterial endothelin does not influence peripheral vascular tone. *J. Cardiovasc. Pharmacol.,* **26** (Suppl. 3), S242–S243.

[30] Karamazyn, M. (1996) The role of endothelin in cardiac function in health and disease. In: *Myocardial Ischemia, Mechanisms, Reperfusion, Protection,* edited by Karamazyn, M., pp. 209–230. Basel, Switzerland, Birkhauser.

[31] Yanagisawa, M., Kurihara, H., Kimura, S., Tomobe, Y., Kobayashi, M., Mitsui, Y., Yazaki, Y., Goto, K., and Masaki, T. (1988) A novel potent vasoconstrictor peptide produced by vascular endothelial cells. *Nature,* **332,** 411–415.

[32] Emori, T., Hirata, Y., Imai, T., Ohta, K., Kanno, K., Eguchi, S., and Marumo, F. (1992) Cellular mechanism of thrombin on endothelin-1 biosynthesis and release in bovine endothelial cell. *Biochem. Pharmacol.,* **44,** 2409–2411.

[33] Kurihara, H., Yoshizumi, M., Sugiyama, T., Takaku, F., Yanagisawa, M., Masaki, T., Hamaoki, M., Kato, H., and Yazaki, Y. (1989) Transforming growth factor-beta stimulates the expression of endothelin mRNA by vascular endothelial cells, *Biochem. Biophys. Res. Commun.,* **159,** 1435–1440.
[34] Malek, A. M., Zhang, J., Jiang, J., Alper, S. L., and Izumo, S. (1999) Endothelin-1 gene suppression by shear stress: Pharmacological evaluation of the role of tyrosine kinase, intracellular calcium, cytoskeleton, and mechanosensitive channels. *J. Mol. Cell. Cardiol.,* **31,** 387–399.
[35] Yoshimoto, S., Ishizaki, Y., Sasaki, T., and Murota, S. (1991) Effect of carbon dioxide and oxygen on endothelin production by cultured porcine cerebral endothelial cells. *Stroke,* **22,** 378–383.
[36] Boulanger, C., and Luscher, T. F. (1990) Release of endothelin from the porcine aorta: Inhibition by endothelium-derived nitric oxide. *J. Clin. Invest.,* **85,** 587–590.
[37] Prins, B. A., Hu, R. M., Nazario, B., Pedram, A., Frank, H. J., Weber, M. A., and Levin, E. R. (1994) Prostaglandin E2 and prostacyclin inhibit the production and secretion of endothelin from cultured endothelial cells. *J. Biol. Chem.,* **269,** 11938–11944.
[38] Kohno, M., Horio, T., Yokokawa, K., Kurihara, N., and Takeda, T. (1992) C-type natriuretic peptide inhibits thrombin- and angiotensin II-stimulated endothelin release via cyclic guanosine 3′,5′-monophosphate. *Hypertension,* **19,** 320–325.
[39] Okumura, K., Nishiura, T., Awaji, Y., Kondo, J., Hashimoto, H., and Ito, T. (1991) 1,2-Diacylglycerol content and its composition in thoracic aorta of diabetic rats. *Diabetes,* **40,** 820–824.
[40] Xia, P., Inoguchi, T., Kern, K. S., Engerman, R. L., Oats, P. J., and King, G. L. (1994) Characterization of the mechanism for the chronic activation of diacylglycerol-protein kinase C in diabetes and hypergalactosaemia. *Diabetes,* **43,** 1122–1129.
[41] Li, W., Wang, W., and Liu, X. (1994) Comparative study of high-glucose effect on phosphatidylcholine hydrolysis of cultured retinal capillary pericytes and endothelial cells. *Biochem. Biophys. Acta,* **1222,** 339–347.
[42] Chen, S., Apostolova, M. D., Cherian, M. G., and Chakrabarti, S. (2000) Interaction of endothelin-1 with vasoactive factors in mediating glucose-induced increased permeability in endothelial cells. *Lab. Invest.,* **80,** 1311–1321.
[43] King, G. L., and Brownlee, M. (1996) The cellular and molecular mechanisms of diabetic complications. *Endocrinol. Metab. Clin. North. Am.,* **25,** 255–270.
[44] Koya, D., and King, G. L. (1998) Protein kinase C activation and the development of diabetic complications. *Diabetes,* **47,** 859–866.
[45] Johannes, F. J., Prestle, J., Eis, S., Oberhagemann, P., and Pfizenmaier, K. (1994) PKC is a novel, atypical member of the protein kinase C family. *J. Biol. Chem.,* **269,** 6140–6148.
[46] Lynch, J. J., Ferro, T. J., Blumenstock, F. A., Brockenauer, A. M., and Malik, A. B. (1990) Increased endothelial albumin permeability mediated by protein kinase C activation. *J. Clin. Invest.,* **85,** 991–998.
[47] Williams, B., Gallacher, B., Patel, H., and Orme, C. (1997) Glucose-induced protein kinase C activation regulates vascular permeability factor mRNA expression and peptide production by human vascular smooth muscle cells in vitro. *Diabetes,* **46,** 1497–1503.
[48] Kern, T. S., and Engerman, R. L. (1994) Comparison of retinal lesions in alloxan-diabetic rats and galactose-fed rats. *Curr. Eye Res.,* **13,** 863–867.
[49] Kowluru, R. A., Kern, T. S., Engerman, R. L., and Armstrong, D. (1996) Abnormalities of retinal metabolism in diabetes or experimental galactosemia. III. Effects of antioxidants. *Diabetes,* **45,** 1233–1237.
[50] Ishii, H., Jirousek, M. R., Koya, D., Takagi, C., Xia, P., Clermont, A., Bursell, S. E., Kern, T. S., Ballas, L. M., Heath, W. F., Stramm, L. E., Feener, E. P., and King, G. L. (1996) Amelioration of vascular dysfunctions in diabetic rats by an oral PKC beta inhibitor. *Science,* **272,** 728–731.
[51] Williamson, J. R., Chang, K., Frangos, M., Hasan, K. S., Ido, Y., Kawamura, T., Nyengaard, J. R., van den Enden, M., Kilo, C., and Tilton, R. G. (1993) Perspectives in diabetes: Hyperglycemic pseudohypoxia and diabetic complications. *Diabetes,* **42,** 801–813.
[52] Costantino, L., Rastelli, G., Vianello, P., Cignarella, G., and Barlocco, D. (1999) Diabetes complications and their potential prevention: Aldose reductase inhibition and other approaches. *Med. Res. Rev.,* **19,** 3–23.
[53] Engerman, R. L., Kern, T. S., and Garment, M. B. (1993) Capillary basement membrane in retina, kidney, and muscle of diabetic dogs and galactosemic dogs and its response to 5 years aldose reductase inhibition. *J. Diabetes Complications,* **7,** 241–245.
[54] Pugliese, G., Tilton, R. G., and Williamson, J. R. (1991) Glucose-induced metabolic imbalances in the pathogenesis of diabetic vascular disease. *Diabetes Metab. Rev.,* **7,** 35–59.
[55] Vlassara, H. (1997) Recent progress in advanced glycation end products and diabetic complications. *Diabetes,* **46** (Suppl. 2), S19–S25.
[56] Vlassara, H. (2001) The AGE-receptor in the pathogenesis of diabetic complications. *Diabetes Metab. Res. Rev.,* **17,** 436–443.
[57] Bierhaus, A., Hofmann, M. A., Ziegler, R., and Nawroth, P. P. (1998) AGEs and their interaction with AGE-receptors in vascular disease and diabetes mellitus. I. The AGE concept. *Cardiovasc. Res.,* **37,** 586–600.
[58] Takagi, Y., Kashiwagi, A., Tanaka, Y., Asahian, T., Kikkawa, R., and Shigeta, Y. (1995) Significance of fructose-induced protein oxidation and formation of advanced glycation end product. *J. Diabetes Complications,* **9,** 89–91.
[59] Curcio, F., Pegorano, I., dello Russo, P., Falleti, E., Perrilla, G., and Ceriello, A. (1995) SOD and GSH inhibit the high glucose induced oxidative damage and the PDGF increase secretion in cultured endothelial cells. *Thromb. Haemost.,* **74,** 969–973.
[60] Tesfamariam, B., and Cohen, R. A. (1992) Free radicals mediate endothelial cell dysfunction caused by elevated glucose. *Am. J. Phsyiol.,* **263,** H321–H326.
[61] Ohishi, K., and Carmines, P. K. (1995) Superoxide dismutase restores the influence of nitric oxide on renal arterioles in diabetes mellitus. *Am. J. Soc. Nephrol.,* **5,** 1559–1566.
[62] Dileepan, K., Sharma, R., Stechschulte, D. J., and Savin, V. J. (1993) Effect of superoxide exposure on albumin permeability of isolated rat glomeruli. *J. Lab. Clin. Med.,* **121,** 797–804.
[63] Vasan, S., Foiles, P. G., and Founds, H. W. (2001) Therapeutic potential of AGE inhibitors and breakers of AGE protein cross-links. *Expert Opin. Invest. Drugs,* **10,** 1977–1987.
[64] Kern, T. S., and Engerman, R. L. (2001) Pharmacological inhibition of diabetic retinopathy: Aminoguanidine and aspirin. *Diabetes,* **50,** 1636–1642.
[65] Bierhaus, A., Chevion, S., Chevion, M., Hofmann, M., Quehenberger, P., Illmer, T., Luther, T., Berentshtein, E.,

Tritschler, H., Muller, M., Wahl, P., Ziegler, R., and Nawroth, P. P. (1997) Advanced glycation end product-induced activation of NF-$\kappa$B is suppressed by alpha-lipoic acid in cultured endothelial cells. *Diabetes,* **46,** 1481–1490.
[66] Quehenberger, P., Bierhaus, A., Fasching, P., Muellner, C., Klevesath, M., Hong, M., Stier, G., Sattler, M., Schleicher, E., Speiser, W., and Nawroth, P. P. (2000) Endothelin 1 transcription is controlled by nuclear factor-kappaB in AGE-stimulated cultured endothelial cells. *Diabetes,* **49,** 1561–1570.
[67] Hattori, Y., Banba, N., Gross, S. S., and Kasai, K. (1999) Glycated serum albumin-induced nitric oxide production in vascular smooth muscle cells by nuclear factor $\kappa$B-dependent transcriptional activation of inducible nitric oxide synthase. *Biochem. Biophys. Res. Commun.,* **259,** 128–132.
[68] Moncada, S. (1992) The L-arginine/nitric oxide pathway. *Acta Physiol. Scand.,* **145,** 201–227.
[69] Bredt, D. S., and Snyder, S. H. (1990) Isolation of nitric oxide synthetase, a calmodulin-requiring enzyme. *Proc. Natl. Acad. Sci. U. S. A.,* **87,** 682–685.
[70] Moncada, S., Palmer, R. M. J., and Higgs, E. A. (1991) Nitric oxide, physiology, pathophysiology, and pharmacology. *Pharm. Rev.,* **43,** 109–142.
[71] Nathan, C. (1992) Nitric oxide as a secretory product of mammalian cells. *FASEB J.,* **6,** 3051–3064.
[72] Lüscher, T. F. (1990) Imbalance of endothelium-derived relaxing and contracting factors. A new concept in hypertension? *Am. J. Hypertens.,* **3,** 317–330.
[73] Cosentino, F., and Luscher, T. F. (2001) Effects of blood pressure and glucose on endothelial function. *Curr. Hypertens. Rep.,* **3,** 79–88.
[74] Cosentino, F., and Luscher, T. F. (1998) Endothelial dysfunction in diabetes mellitus, *J. Cardiovasc. Pharmacol.,* **32** (Suppl. 3), S54–S61.
[75] Cosentino, F., Hishikawa, K., Katusic, Z. S., and Luscher, T. F. (1997) High glucose increases nitric oxide synthase expression and superoxide anion generation in human aortic endothelial cells. *Circulation,* **96,** 25–28.
[76] van Dam, B., Demirci, C., Reitsma, H. J., van Lambalgen, A. A., van den Bos, G. C., Tangelder, G. J., and Stehouwer, C. D. (2000) Alterations in nitric oxide activity and sensitivity in early streptozotocin-induced diabetes depend on arteriolar size. *Int. J. Exp. Diabetes Res.,* **1,** 221–232.
[77] Stehouwer, C. D., Lambert, J., Donker, A. J., and van Hinsbergh, V. W. (1997) Endothelial dysfunction and pathogenesis of diabetic angiopathy. *Cardiovasc Res.,* **34,** 55–68.
[78] Hunt, J. V., Dean, and R. T., and Wolff, S. P. (1988) Hydroxyl radical production and autoxidative glycosylation. Glucose autoxidation as the cause of protein damage in the experimental glycation model of diabetes mellitus and ageing. *Biochem. J.,* **256,** 205–212.
[79] Nadler, J., and Winer, L. (1996) Free radicals, nitric oxide and diabetic complications. In: *Diabetes Mellitus,* edited by LeRoith, D., Taylor, S. I., and Olefsky, M., pp. 840–848. Philadelphia, Lippincott-Raven.
[80] Jiang, Z. Y., Woollard, A. C., and Wolff, S. P. (1990) Hydrogen peroxide production during experimental protein glycation. *FEBS Lett.,* **268,** 69–71.
[81] Brownlee, M. (2001) Biochemistry and molecular cell biology of diabetic complications. *Nature,* **414,** 813–820.
[82] Nishikawa, T., Edelstein, D., Du, X. L., Yamagishi, S., Matsumura, T., Kaneda, Y., Yorek, M. A., Beebe, D., Oates, P. J., Hammes, H. P., Giardino, I., and Brownlee, M. (2000) Normalizing mitochondrial superoxide production blocks three pathways of hyperglycaemic damage. *Nature,* **404,** 787–790.
[83] Du, X. L., Edelstein, D., Dimmeler, S., Ju, Q., Sui, C., and Brownlee, M. (2001) Hyperglycemia inhibits endothelial nitric oxide synthase activity by posttranslational modification at the Akt site. *J. Clin. Invest.,* **108,** 1341–1348.
[84] Craven, P. A., Phillips, S. L., Melhem, M. F., Liachenko, J., and DeRubertis, F. R. (2001) Overexpression of manganese superoxide dismutase suppresses increases in collagen accumulation induced by culture of mesangial cells in high-media glucose. *Metabolism,* **50,** 1043–1048.
[85] Hammes, H. P., Lin, J., Bretzel, R. G., Brownlee, M., and Breier, G. (1998) Upregulation of the vascular endothelial growth factor/vascular endothelial growth factor receptor system in experimental background diabetic retinopathy of the rat. *Diabetes,* **47,** 401–406.
[86] Pe'er, J., Folberg, R., Itin, A., Gnessin, H., Hemo, I., and Keshet, E. (1996) Upregulated expression of vascular endothelial growth factor in proliferative diabetic retinopathy. *Br. J. Ophthalmol.,* **80,** 241–245.
[87] Joussen, A. M., Huang, S., Poulaki, V., Camphausen, K., Beecken, W. D., Kirchhof, B., and Adamis, A. P. (2001) In vivo retinal gene expression in early diabetes. *Invest. Ophthalmol. Vis. Sci.,* **42,** 3047–3057.
[88] de Vriese, A. S., Tilton, R. G., Elger, M., Stephan, C. C., Kriz, W., and Lameire, N. H. (2001) Antibodies against vascular endothelial growth factor improve early renal dysfunction in experimental diabetes. *J. Am. Soc. Nephrol.,* **12,** 993–1000.
[89] Aiello, L. P., Bursell, S. E., Clermont, A., Duh, E., Ishii, H., Takagi, C., Mori, F., Ciulla, T. A., Ways, K., Jirousek, M., Smith, L. E. H., and King, G. L. (1997) Vascular endothelial growth factor-induced retinal permeability is mediated by protein kinase C in vivo and suppressed by an orally effective $\beta$-isoform-selective inhibitor, *Diabetes,* **46,** 1473–1480.
[90] Qaum, T., Xu, Q., Joussen, A. M., Clemens, M. W., Qin, W., Miyamoto, K., Hassessian, H., Wiegand, S. J., Rudge, J., Yancopoulos, G. D., and Adamis, A. P. (2001) VEGF-initiated blood- retinal barrier breakdown in early diabetes. *Invest. Ophthalmol. Vis. Sci.,* **42,** 2408–2413.
[91] Leung, D. W., Cachianes, G., Kuang, W. J., Goeddel, D. V., and Ferrara, N. (1989) Vascular endothelial growth factor is a secreted angiogenic mitogen. *Science,* **246,** 1306–1309.
[92] Tischer, E., Mitchell, R., Hartman, T., Silva, M., Gospodarowicz, D., Fiddes, J. C., and Abraham, J. A. (1991) The human gene for vascular endothelial growth factor. Multiple protein forms are encoded through alternative exon splicing. *J. Biol. Chem.,* **266,** 11947–11954.
[93] Kato, H., Suzuki, H., Tajima, S., Ogata, Y., Tominaga, T., Sato, A., and Saruta, T. (1991) Angiotensin II stimulates collagen synthesis in cultured vascular smooth muscle cells. *J Hypertens.,* **9,** 17–22.
[94] Rabelink, T. J., and Bakris, G. L. (1998) The renin-angiotensin system in diabetic nephropathy: The endothelial connection. *Mineral Electrolyte Metab.,* **24,** 381–388.
[95] Moreau, P., d'Uscio, L. V., Shaw, S., Takase, H., Barton, M., and Luscher, T. F. (1997) Angiotensin II increases tissue endothelin and induces vascular hypertrophy: Reversal by ET(A)-receptor antagonist. *Circulation,* **96,** 1593–1597.

[96] Ferri, C., Pittoni, V., Piccoli, A., Laurenti, O., Cassone, M. R., Bellini, C., Properzi, G., Valesini, G., De Mattia, G., and Santucci, A. (1995) Insulin stimulates endothelin-1 secretion from human endothelial cells and modulates its circulating levels in vivo. *J. Clin. Endocrinol. Metab.,* **80,** 829–835.

[97] Chakrabarti, S., and Sima, A. A. (1997) Endothelin-1 and endothelin-3-like immunoreactivity in the eyes of diabetic and non-diabetic BB/W rats. *Diabetes Res. Clin. Pract.,* **37,** 109–120.

[98] Hopfner, R. L., Misurski, D., Wilson, T. W., McNeill, J. R., and Gopalakrishnan, V. (1998) Insulin and vanadate restore decreased plasma endothelin concentration and exaggerated vascular responses to normal in streptozotocin diabetic rat. *Diabetologia,* **41,** 1233–1240.

[99] Hargrove, G. M., Dufresne, J., Whiteside, C., Muruve, D. A., and Wong, N. C. (2000) Diabetes mellitus increases endothelin-1 gene transcription in rat kidney. *Kidney Int.,* **58,** 1534–1545.

[100] Chen, S., Evans, T., Mukherjee, K., Karmazyn, M., and Chakrabarti, S. (2000) Diabetes-induced myocardial structural changes: role of endothelin-1 and its receptors. *J. Mol. Cell. Cardiol.,* **32,** 1621–1629.

[101] Deng, D., Evans, T., Mukherjee, K., Downey, D., and Chakrabarti, S. (1999) Diabetes-induced vascular dysfunction in the retina: Role of endothelins. *Diabetologia,* **42,** 1228–1234.

[102] Chakravarthy, U., Hayes, R. G., Stitt, A. W., and Douglas, A. (1997) Endothelin expression in ocular tissues of diabetic and insulin-treated rats. *Invest. Ophthalmol. Vis. Sci.,* **38,** 2144–2151.

[103] Wu, S. Q., and Tang, F. (1998) Impaired paracrine effect of endothelin-1 on vascular smooth muscle in streptozotocin-diabetic rats. *Cardiovasc. Res.,* **39,** 651–656.

[104] Cameron, N. E., Dines, K. C., and Cotter, M. A. (1994) The potential contribution of endothelin-1 to neurovascular abnormalities in streptozotocin-diabetic rats. *Diabetologia,* **37,** 1209–1215.

[105] Letizia, C., Iannaccone, A., Cerci, S., Santi, G., Cilli, M., Coassin, S., Pannarale, M. R., Scavo, D., and Iannacone, A. (1997) Circulating endothelin-1 in non-insulin-dependent diabetic patients with retinopathy. *Horm. Metab. Res.,* **29,** 247–251.

[106] Laurenti, O., Vingolo, E. M., Desideri, G. B., Ferri, C., Bellini, C., Cassone-Faldetta, M., Santucci, A., and De Mattia, G. (1997) Increased levels of plasma endothelin-1 in non-insulin dependent diabetic patients with retinopathy but without other diabetes-related organ damage. *Exp. Clin. Endocrinol. Diabetes,* **105** (Suppl. 2), 40–42.

[107] De Mattia, G., Cassone-Faldetta, M., Bellini, C., Bravi, M. C., Laurenti, O., Baldoncini, R., Santucci, A., and Ferri, C. (1998) Role of plasma and urinary endothelin-1 in early diabetic and hypertensive nephropathy. *Am. J. Hypertens.,* **11,** 983–988.

[108] Takahashi, K., Ghatei, M. A., Lam, H. C., O'Halloran, D. J., and Bloom, S. R. (1990) Elevated plasma endothelin in patients with diabetes mellitus. *Diabetologia,* **33,** 306–310.

[109] Morise, T., Takeuchi, Y., Kawano, M., Koni, I., and Takeda, R. (1995) Increased plasma levels of immunoreactive endothelin and von Willebrand factor in NIDDM patients. *Diabetes Care,* **18,** 87–89.

[110] Kawamura, M., Ohgawara, H., Naruse, M., Suzuki, N., Iwasaki, N., Naruse, K., Hori, S., Demura, H., and Omori, Y. (1992) Increased plasma endothelin in NIDDM patients with retinopathy. *Diabetes Care,* **15,** 1396–1397.

[111] Donatelli, M., Colletti, I., Bucalo, M. L., Russo, V., and Verga, S. (1994) Plasma endothelin levels in NIDDM patients with macroangiopathy. *Diabetes Res.,* **25,** 159–164.

[112] Kamoi, K., Ishibashi, M., and Yamaji, T. (1994) Endothelin-1 and big endothelin-1 in NIDDM patients with and without microangiopathy. *Diabetes Res. Clin. Pract.,* **24,** 125–129.

[113] Bertello, P., Veglio, F., Pinna, G., Gurioli, L., Molino, P., Alban, S., and Chiandussi, L. (1994) Plasma endothelin in NIDDM patients with and without complications. *Diabetes Care,* **17,** 574–577.

[114] Haak, T., Jungmann, E., Felber, A., Hillmann, U., and Usadel, K. H. (1992) Increased plasma levels of endothelin in diabetic patients with hypertension. *Am. J. Hypertens.,* **5,** 161–166.

[115] Rubanyi, G. M., and Polokof, M. A. (1994) Endothelins: Molecular biology, biochemistry, pharmacology, physiology and pathophysiology. *Pharmacology Rev.,* **46,** 326–414.

[116] Levin, E. R. (1995) Endothelins. *N. Engl. J. Med.,* **333,** 356–363.

[117] Jorkasky, D. K., Hay, D. W. P., and Freed, M. I. (1995) The role of endothelin in human disease: Implications and potential therapeutic intervention. In: *Endothelin Receptors: From the Genes to the Human,* edited by Ruffolo, R. R., Jr., pp. 215–217. Boca Raton, FL, CRC Press.

[118] Chen, S., Mukherjee, S., and Chakrabarti, S. (2001) Hyperglycemia induced, endothelin (ET) mediated, increased extracellular matrix (ECM) protein synthesis is mediated via NFKB activation. *Diabetes,* **50** (Suppl. 2), A191.

[119] Evans, T., Deng, D. X., Chen, S., and Chakrabarti, S. (2000) Endothelin receptor blockade prevents augmented extracellular matrix component mRNA expression and capillary basement membrane thickening in the retina of diabetic and galactose-fed rats. *Diabetes,* **49,** 662–666.

[120] Salani, D., Taraboletti, G., Rosano, L., Di Castro, V., Borsotti, P., Giavazzi, R., and Bagnato, A. (2000) Endothelin-1 induces an angiogenic phenotype in cultured endothelial cells and stimulates neovascularization in vivo. *Am. J. Pathol.,* **157,** 1703–1711.

[121] Bek, E. L., and McMillen, M. A. (2000) Endothelins are angiogenic. *J. Cardiovasc. Pharmacol.,* **36** (5 Suppl. 1), S135–S139.

[122] Brennan, P. A., and Zaki, G. A. (2000) Angiogenesis in cancer: The role of endothelin-1. *Ann. R. Coll. Surg. Engl.,* **82,** 363–364.

[123] Caird, F. I. (1971) The epidemiology of diabetic microangiopathy. *Acta Diabetol. Lat.,* **8** (Suppl. 1), 240–248.

[124] Mazze, R. S., Sinnock, P., Deeb, L., and Brimberry, J. L. (1985) An epidemiological model for diabetes mellitus in the United States: Five major complications. *Diabetes Res. Clin. Pract.,* **1,** 185–191.

[125] Chakrabarti, S., Cukiernik, M., Hileeto, D., Evans, T., and Chen, S. (2000) Role of vasoactive factors in the pathogenesis of early changes in diabetic retinopathy. *Diabetes Metab. Res. Rev.,* **16,** 393–407.

[126] Lorenzi, M., and Gerhardinger, C. (2001) Early cellular and molecular changes induced by diabetes in the retina. *Diabetologia,* **44,** 791–804.

[127] Malamitsi-Puchner, A., Economou, E., Katsouyanni, K., Karachaliou, F., Delis, D., and Bartsocas, C. S. (1996) Endothelin 1-21 plasma concentrations in children and adolescents with insulin-dependent diabetes mellitus. *J. Pediatr. Endocrinol. Metab.,* **9,** 463–468.

[128] Smulders, R. A., Stehouwer, C. D., Olthof, C. G., van Kamp, G. J., Teerlink, T., de Vries, P. M., and Donker, A. J. (1994) Plasma endothelin levels and vascular effects of intravenous L-arginine infusion in subjects with uncomplicated insulin-dependent diabetes mellitus. *Clin. Sci.,* **87,** 37–43.

[129] Collier, A., Leach, J. P., McLellan, A., Jardine, A., Morton, J. J., and Small, M. (1992) Plasma endothelinlike immunoreactivity levels in IDDM patients with microalbuminuria, *Diabetes Care,* **15,** 1038–1040.

[130] Ak, G., Buyukberber, S., Sevinc, A., Turk, H. M., Ates, M., Sari, R., Savli, H., and Cigli, A. (2001) The relation between plasma endothelin-1 levels and metabolic control, risk factors, treatment modalities, and diabetic microangiopathy in patients with Type 2 diabetes mellitus. *J. Diabetes Complications,* **15,** 150–157.

[131] Guvener, N., Aytemir, K., Aksoyek, S., and Gedik, O. (1997) Plasma endothelin-1 levels in non-insulin dependent diabetes mellitus patients with macrovascular disease. *Coron. Artery Dis.,* **8,** 253–258.

[132] Kanno, K., Hirata, Y., Shichiri, M., and Marumo, F. (1991) Plasma endothelin-1 levels in patients with diabetes mellitus with or without vascular complication. *J. Cardiovasc. Pharmacol.,* **17** (Suppl. 7), S475–S476.

[133] Fernandez, N., Garcia-Villalon, A. L., Borbujo, J., Monge, L., Garcia, J. L., Gomez, B., and Dieguez, G. (1994) Cooling effects on the histaminergic response of rabbit ear and femoral arteries: Role of the endothelium. *Acta Physiol. Scand.,* **151,** 441–451.

[134] Perfetto, F., Tarquini, R., de Leonardis, V., Piluso, A., Lombardi, V., and Tarquini, B. (1995) Angiopathy affects circulating endothelin-1 levels in type 2 diabetic patients. *Acta Diabetol.,* **32,** 263–267.

[135] Ripodas, A., de Juan, J. A., Roldan-Pallares, M., Bernal, R., Moya, J., Chao, M., Lopez, A., Fernandez-Cruz, A., and Fernandez-Durango, R. (2001) Localisation of endothelin-1 mRNA expression and immunoreactivity in the retina and optic nerve from human and porcine eye: Evidence for endothelin-1 expression in astrocytes. *Brain Res.,* **912,** 137–143.

[136] Park, J. Y., Takahara, N., Gabriele, A., Chou, E., Naruse, K., Suzuma, K., Yamauchi, T., Ha, S. W., Meier, M., Rhodes, C. J., and King, G. L. (2000) Induction of endothelin-1 expression by glucose: An effect of protein kinase C activation. *Diabetes,* **49,** 1239–1248.

[137] Chakrabarti, S., Gan, X. T., Merry, A., Karmazyn, M., and Sima, A. A. (1998) Augmented retinal endothelin-1, endothelin-3, endothelinA and endothelinB gene expression in chronic diabetes. *Curr. Eye Res.,* **17,** 301–307.

[138] De Juan, J. A., Moya, F. J., Ripodas, A., Bernal, R., Fernandez-Cruz, A., and Fernandez-Durango, R. (2000) Changes in the density and localisation of endothelin receptors in the early stages of rat diabetic retinopathy and the effect of insulin treatment. *Diabetologia,* **43,** 773–785.

[139] Takagi, C., Bursell, S. E., Lin, Y. W., Takagi, H., Duh, E., Jiang, Z., Clermont, A. C., and King, G. L. (1996) Regulation of retinal hemodynamics in diabetic rats by increased expression and action of endothelin-1. *Invest. Ophthalmol. Vis. Sci.,* **37,** 2504–2518.

[140] Lippton, H. L., Hauth, T. A., Summer, W. R., and Hyman, A. L. (1989) Endothelin produces pulmonary vasoconstriction and systemic vasodilation. *J. Appl. Physiol.,* **66,** 1008–1012.

[141] Luscher, T. F. (1990) Imbalance of endothelium-derived relaxing and contracting factors: A new concept in hypertension? *Am. J. Hypertens.,* **3,** 317–330.

[142] Seo, B., Oemar, B. S., Siebenmann, R., von Segesser, L., and Luscher, T. F. (1994) Both ETA and ETB receptors mediate contraction to endothelin-1 in human blood vessels. *Circulation,* **89,** 1203–1208.

[143] Warner, T. D., de Nucci, G., and Vane, J. R. (1989) Rat endothelin is a vasodilator in the isolated perfused mesentery of the rat. *Eur. J. Pharmacol.,* **159,** 325–326.

[144] Wright, C. E., and Fozard, J. R. (1988) Regional vasodilation is a prominent feature of the haemodynamic response to endothelin in anaesthetized, spontaneously hypertensive rats. *Eur. J. Pharmacol.,* **155,** 201–203.

[145] de Nucci, G., Thomas, R., D'Orleans-Juste, P., Antunes, E., Walder, C., Warner, T. D., and Vane, J. R. (1988) Pressor effects of circulating endothelin are limited by its removal in the pulmonary circulation and by the release of prostacyclin and endothelium-derived relaxing factor. *Proc. Natl. Acad. Sci. U.S.A.,* **85,** 9797–9800.

[146] Dohi, Y., and Luscher, T. F. (1991) Endothelin in hypertensive resistance arteries. Intraluminal and extraluminal dysfunction. *Hypertension,* **18,** 543–549.

[147] Rae, G. A., Trybulec, M., de Nucci, G., and Vane, J. R. (1989) Endothelin-1 releases eicosanoids from rabbit isolated perfused kidney and spleen. *J. Cardiovasc. Pharmacol.,* **13** (Suppl. 5), S89–S92.

[148] Vane, J. (1990) Endothelins come home to roost. *Nature,* **348,** 673.

[149] Apostolova, M. D., Chen, S., Chakrabarti, S., and Cherian, M. G. (2001) High-glucose-induced metallothionein expression in endothelial cells: An endothelin-mediated mechanism. *Am. J. Physiol. Cell. Physiol.,* **281,** C899–C907.

[150] Yanagisawa, M., Inoue, A., Ishikawa, T., Kasuya, Y., Kimura, S., Kumagaye, S., Nakajima, K., Watanabe, T. X., Sakakibara, S., and Goto, K. (1988) Primary structure, synthesis, and biological activity of rat endothelin, an endothelium-derived vasoconstrictor peptide. *Proc. Natl. Acad. Sci. U. S. A.,* **85,** 6964–6947.

[151] Gonzalez, W., Chen, Z., and Damon, D. H. (2001) Transforming growth factor-beta regulation of endothelin expression in rat vascular cell and organ cultures. *J. Cardiovasc. Pharmacol.,* **37,** 219–226.

[152] Cukiernik, M., Evans, T., Hileeto, D., Downey, D., and Chakrabarti, S. (2000) Interaction of endothelins and nitric oxide synthase in the retina of streptozotocin induced diabetic rats in mediating vasoconstriction. *Can. J. Diabetes Care,* **24** (Suppl. 1), A35.

[153] Cukiernik, M., Hileeto, D., Evans, T., Karmazyn, M., Downey, D., and Chakrabarti, S. (2001) The role of the sodium/hydrogen exchanger-1 in the pathogenesis of diabetic retinal microangiopathy. *Diabetes,* **50** (Suppl. 2), A17 (Abstract).

[154] Oku, H., Kida, T., Sugiyama, T., Hamada, J., Sato, B., and Ikeda, T. (2001) Possible involvement of endothelin-1 and nitric oxide in the pathogenesis of proliferative diabetic retinopathy. *Retina,* **21,** 674–651.

[155] Held, P. J. , Port, F. K., Blagg, C. R., and Agoda, L. Y. C. (1990) United States Renal Data System 1990 annual report. *Am. J. Kidney Dis.,* **2** (Suppl.), 1–106.

[156] Andersen, A. R., Christiansen, J. S., Anderson, J. K., Kreiner, S., and Dekertt, T. (1983) Diabetic neuropathy in type I (insulin-dependent) diabetes: An epidemiological study. *Diabetologia,* **25,** 496–501.

[157] Breyer, J. A., Bain, R. P., Evans, J. K. Nahman, N. S., Jr., Lewis, E. J., Cooper, M., McGill, J., and Berl, T. (1996) Predictors of the progression of renal insufficiency in patients with insulin-dependent diabetes and overt diabetic nephropathy. *Kidney Int.,* **50,** 1651–1658.

[158] Mogensen, C. E., Christensen, C. K., and Vittinghus, E. (1983) The stages in diabetic renal disease: With emphasis in the stage of incipient diabetic neuropathy. *Diabetes,* **32,** 64–78.

[159] Cotran, R. S., Kumar, V., and Collin, S. (1999) The kidney. In: *Robbins Pathologic Basis of Disease,* 6th ed., edited by Cotran, R. S., Kumar, V., and Collins, T., pp. 966–968. Toronto, Canada, WB Sanders.

[160] Steffes, M., Pluth, R., Schimidt, D., McCrery, R., Basgen, J., and Mauer, M. (1996) Glomerular and mesangial cell number in insulin dependent diabetes mellitus (IDDM). In: *Proceedings of the European Diabetic Nephropathy Study Group,* 9th Meeting, Parma, Italy, p. 27.

[161] Epstein, F. H. (1998) Pathophysiology of progressive nephropathies. *N. Engl. J. Med.,* **339,** 1448–1456.

[162] United Kingdom Prospective Diabetic Study. (1998) Intense blood-glucose control with sulphonylureas or insulin compared with conventional treatment and risk of complications in patients with type 2 diabetes (UKPDS 33). *Lancet,* **352,** 837–853.

[163] Diabetes Control and Compilations Research Group. (1995) Effect of intensive therapy. *Kidney Int.,* **47,** 1703–1720.

[164] Simonson, M. S. (1993) Endothelins: Multifunctional renal peptides. *Physiol. Rev.,* **73,** 375–411.

[165] Chen, S., Evans, T., Deng, D., Cukiernik, M., and Chakrabarti, S. (2002) Hyperhexosemia induced functional and structural changes in the kidneys: Role of endothelins. *Nephron,* **90,** 86–94.

[166] Turner, N. C., Morgan, P. J., Haynes, A. C., Vidgeon-Hart, M., Toseland, N., and Clapham, J. C. (1997) Elevated renal endothelin-1 clearance and mRNA levels associated with albuminuria and nephropathy in non-insulin dependent diabetes mellitus studies in obese fa/fa Zucker rats. *Clin. Sci. (Colch.),* **93,** 565–571.

[167] Khan, M. A., Dashwood, M. R., Thompson, C. S., Mumtaz, F. H., Mikhailidis, D. P., and Morgan, R. J. (1999) Time-dependent up-regulation of endothelin-A receptors and down-regulation of endothelin-B receptors and nitric oxide synthase binding sites in the renal medulla of a rabbit model of partial bladder outlet obstruction: Potential clinical relevance. *BJU Int.,* **84,** 1073–1080.

[168] Ishimura, E., Shouji, S., Nishizawa, Y., Morii, H., and Kashgarian, M. (1991) Regulation of mRNA expression for ECM by cultured rat mesangial cells. *J. Am. Soc. Nephrol.,* **2,** 546.

[169] Nakamura, T., Ebihara, I., Fukui, M., Tomino, Y., and Koida, H. (1995) Effect of a specific endothelin-A receptor antagonist on mRNA levels for extracellular matrix components and growth factors in diabetic glomeruli. *Diabetes,* **44,** 895–899.

[170] Koya, D., Jirousek, M. R., Lin, Y. W., Ishii, H., Kuboki, K., and King, G. L. (1997) Characterization of protein kinase C beta isoform activation on the gene expression of transforming growth factor-beta, extracellular matrix components, and prostanoids in the glomeruli of diabetic rats. *J. Clin. Invest.,* **100,** 115–126.

[171] Boulton, A. J. M. (1993) Pathogenesis of diabetic neuropathy. In: *The Diabetes Annual,* edited by Marshall, S. M., Home, P. D., Alberti, K. G. M. M., and Krall, L. P., **11,** p. 192. Amsterdam, The Netherlands, Elsevier Science Publishers BV.

[172] Thomas, P. K. (1992) Diabetic neuropathy: Models, mechanisms and mayhem. *Can. J. Neurol. Sci.,* **19,** 1.

[173] Sima, A. A. F., and Sugimoto, K. (1999) Experimental diabetic neuropathy: An update. *Diabetologia,* **42,** 773–788.

[174] Zhu, X., and Eichberg, J. (1990) 1,2-Diacylglycerol content and its arachidonyl-containing molecular species are reduced in sciatic nerve from streptozotocin-induced diabetic rats. *J. Neurochem.,* **55,** 1087–1090.

[175] Cameron, N. E., Eaton, S. E. M., Cotter, M. A., and Tesfaye, S. (2001) Vascular factors and metabolic interactions in the pathogenesis of diabetic neuropathy. *Diabetologia,* **44,** 1973–1988.

[176] Hermenegildo, C., Felipo, V., Minana, M. D., Romero, F. J., and Grisolia, S. (1993) Sustained recovery of $Na^{+}/K^{+}$-ATPase activity in sciatic nerve of diabetic mice by administration of H7 or callphostin C, inhibitors of protein kinase C. *Diabetes,* **42,** 257.

[177] Catalan, R. E., Martinez, A. M., Aragones, M. D., Fernandez, I., Miguel, B. G., Perez, M. J., and Calcerrada, M. C. (1996) Endothelin-stimulated phosphoinositide turnover and protein kinase C translocation in rat synaptosomes. *Biochem. Mol. Biol. Int.,* **38,** 7–14.

[178] Cameron, N. E., and Cotter, M. A. (1996) Rapid reversal of by aminoguanidine of the neurovascular effects of diabetes in rats: Modulation by nitric oxide synthase inhibition. *Metabolism,* **45,** 1147–1152.

[179] Stevens, E. J., and Tomlinson, D. R. (1995) Effects of endothelin receptor antagonism with bosentan on peripheral nerve function in experimental diabetes. *Br. J. Pharmacol.,* **115,** 373–379.

[180] Deng, D., Evans, T., Xu, G., Sima, A. A. F., and Chakrabarti, S. (1998) Endothelin-1 and its receptors in the pathogenesis of neuronal and retinal functional abnormalities in the diabetic rats. *Diabetes,* **47** (Suppl. 1), A142.

[181] Zochodone, D. W., Cheng, C., and Sun, H. (1996) Diabetes increases sciatic nerve susceptibility to endothelin induced ischemia. *Diabetes,* **45,** 627–632.

[182] Haffner, S. M., Lehto, S., Rönnemaa, T., Pyörälä, K., and Laakso, M. (1998) Mortality from coronary heart disease in subjects with type 2 diabetes and in nondiabetic subjects with and without prior myocardial infarction. *N. Engl. J. Med.,* **339,** 229–234.

[183] Frost, D., and Beischer, W. (1998) Determinants of carotid artery wall thickening in young patients with type 1 diabetes mellitus. *Diabet Med.,* **15,** 851–857.

[184] Hamalainen, H., Ronnemaa, T., Halonen, J. P., and Toikka, T. (1999) Factors predicting lower extremity amputations in patients with type 1 or type 2 diabetes mellitus: A population-based 7-year follow-up study. *J. Intern. Med.,* **246,** 97–103.

[185] Jandeleit-Dahm, K., Allen, T. J., Youssef, S., Gilbert, R. E., and Cooper, M. E. (2000) Is there a role for endothelin antagonists in diabetic renal diseases? *Diabetes Obesity Metab.,* **2,** 15–24.

[186] Jimenez, M., Daret, D., Choussat, A., and Bonnet, J. (1999) Related articles immunohistological and ultrastructural analysis of the intimal thickening in coarctation of human aorta. *Cardiovasc. Res.,* **41,** 737–745.

[187] Hopfner, R. L., McNeill, J. R., and Gopalakrishnan, V. (1999) Plasma endothelin level and vascular responses at different temporal stages of streptozotocin diabetes. *Eur. J. Pharmacol.,* **374,** 221–227.

[188] Takeda, Y., Miyamori, I., Yoneda, T., and Takeda, R. (1991) Production of endothelin-1 from the mesenteric arteries of streptozotocin-induced diabetic rats. *Life Sci.,* **48,** 2553–2556.

[189] Perfet, F., Tarquini, R., Tapparini, L., and Tarquini, B. (1998) Influence of non-insulin-dependent diabetes mellitus on plasma endothelin-1 levels in patients with advanced atherosclerosis. *J. Diabetes Complications,* **12,** 187–192.

[190] Gilbert, R. E., Rumber, J. R., Cao, Z. M., Cox, A. J., van Eeden, P., Allen, T. J., Kelly, D. J., and Cooper, M. E. (2000) Endothelin receptor antagonism ameliorates mast cell infiltration, vascular hypertrophy and epidermal growth factor expression in experimental diabetes. *Circ. Res.,* **86,** 158–165.

[191] Migdalis, I. N., Kalogeropoulou, K., Iliopoulou, V., Triantafilou, P., Charalabides, J., Mortzos, G., and Cordopatis, C. (2001) Plasma levels of endothelin, lipid peroxides and prostacyclin in diabetic patients with macroangiopathy. *Diabetes Res. Clin. Pract.,* **54,** 129–136.

[192] Makino, A., Oda, S., and Kamata, K. (2001) Mechanisms underlying increased release of endothelin-1 from aorta in diabetic rats. *Peptides,* **22,** 639–645.

[193] Makino, A., and Kamata, K. (1998) Elevated plasma endothelin-1 levels in streptozotocin induced diabetic rats and responsiveness of the mesenteric arterial bed to endothelin-1. *Br. J. Pharmacol.,* **123,** 1065–1072.

[194] Moreau, P., d'Uscio, L. V., Shaw, S., Takase, H., Barton, M., and Luscher, T. F. (1997) Angiotensin II increases tissue endothelin and induces vascular hypertrophy: Reversal by ET(A)-receptor antagonist. *Circulation,* **96,** 1593–1597.

[195] Shehadeh, A., and Regan, T. J. (1995) Cardiac consequences of diabetes mellitus. *Clin. Cardiol.,* **18,** 301–305.

[196] Lewinter, M. M. (1996) Diabetic cardiomyopathy: An overview. *Coron Artery Dis.,* **7,** 95–98.

[197] Nunoda, S., Genda, A., Sekiguchi, M., and Takeda, R. (1985) Left ventricular endomyocardial biopsy findings in patients with essential hypertension and hypertrophic cardiomyopathy with special reference to the incidence of bizarre myocardial hypertrophy with disorganization and biopsy score. *Heart Vessels,* **1,** 170–175.

[198] Savage, M. P., Krolewski, A. S., Kenien, G. G., Lebeis, M. P., Christlieb, A. R., and Lewis, S. M. (1988) Acute myocardial infarction in diabetes mellitus and significance of congestive heart failure as a prognostic factor. *Am. J. Cardiol.,* **62,** 665–669.

[199] Suzuki, T., Kumazaki, T., and Mitsui, Y. (1993) Endothelin-1 is produced and secreted by neonatal rat cardiac myocytes in vitro. *Biochem. Biophys. Res. Commun.,* **191,** 823–830.

[200] Chua, B. H., Chua, C. C., Diglio, C. A., and Siu, B. B. (1993) Regulation of endothelin-1 mRNA by angiotensin II in rat heart endothelial cells. *Biochem. Biophys. Acta,* **1178,** 201–206.

[201] Fukuchi, M., and Giaid, A. (1998) Expression of endothelin-1 and endothelin-converting enzyme-1 mRNAs and proteins in failing human hearts. *J. Cardiovasc. Pharmacol.,* **31** (Suppl. 1), S421–S423.

[202] Ganong, W. (1997) Cardiovascular regulatory mechanisms: Endothelins, In: *Medical Physiology,* pp. 555–556. Stamford, CT, Appleton and Lang.

[203] Hu, J. R., von Harsdorf, R., and Lang, R. E. (1988) Endothelin has potent inotropic effects in rat atria. *Eur. J. Pharmacol.,* **158,** 275–278.

[204] Ishikawa, T., Yanagisawa, M., Kimura, S., Goto, K., and Masaki, T. (1988) Positive chronotropic effects of endothelin, a novel endothelium-derived vasoconstrictor peptide. *Pflugers Arch.,* **413,** 108–110.

[205] Lieu, A. T., and Reid, J. J. (1994) Changes in the responsiveness to endothelin-1 in isolated atria from diabetic rats. *Eur. J. Pharmacol.,* **261,** 33–42.

[206] Vesci, L., Mattera, G. G., Tobia, P., Corsico, N., and Calvani, M. (1995) Cardiac and renal endothelin-1 binding sites in streptozotocin-induced diabetic rats. *Pharmacol. Res.,* **32,** 363–367.

[207] Erbas, T., Erbas, B., Kabakci, G., Aksoyek, S., Koray, Z., and Gedik, O. (2000) Plasma big-endothelin levels, cardiac autonomic neuropathy, and cardiac functions in patients with insulin-dependent diabetes mellitus. *Clin. Cardiol.,* **23,** 259–263.

[208] Sharma, A. C., Fogelson, B. G., Nawas, S. I., Vigneswaran, W. T., Sam, A. D., 2nd, Alden, K. J., Ferguson, J. L., and Law, W. R. (1999) Elevated coronary endothelin-1 but not nitric oxide in diabetics during CABG. *Ann. Thorac. Surg.,* **67,** 1659–1663.

[209] Karmazyn, M., Gan, X. T., Humphreys, R. A., Yoshida, H., and Kusumoto, K. (1999). The myocardial Na(+)-H(+) exchange: Structure, regulation and its role in heart disease, *Circ. Res.,* **85,** 777–786.

[210] Lazdunski, M., Frelin, C., and Vigne, P. (1985) The sodium/hydrogen exchange system in cardiac cells: Its biochemical and pharmacological properties and its role in regulating internal concentrations of sodium and internal pH. *J. Mol. Cell. Cardiol.,* **17,** 1029–1042.

[211] Hileeto, D., Cukiernik, M., Evans, T., Chen, S., Mukherjee, S., Downey, D., Karmazyn, M., and Chakrabarti, S. (2001) Interaction of endothelin-1 and sodium/hydrogen exchanger-1 in the pathogenesis of diabetes induced changes in the heart. *Diabetes,* **50** (Suppl. 2), A155 (Abstract).

[212] Vanhoutte, P. M. (1994) Endothelin-1: A matter of life and breath. *Nature,* **368,** 693–694.

[213] Matsuura, A., Kawashima, S., Yamochi, W., Hirata, K., Yamaguchi, T., Emoto, N., and Yokoyama, M. (1997) Vascular endothelial growth factor increases endothelin-converting enzyme expression in vascular endothelial cells. *Biochem. Biophys. Res. Commun.,* **235,** 713–716.

[214] Pedram, A., Razandi, M., Hu, R. M., and Levin, E. R. (1997) Vasoactive peptides modulate vascular endothelial cell growth factor production and endothelial cell proliferation and invasion. *J. Biol. Chem.,* **27,** 17097–17103.

[215] Mukherjee, S., Evans, T., Chen, S., Cukiernik, M., Hileeto, D., Karmazyn, M., and Chakrabarti, S. (2001) Nitric oxide in diabetic hearts. *Can. J. Diabetes Care,* **25,** 192.

[216] Sorbinil Retinopathy Trial Research Group. (1990) A randomized trial of sorbinil, an aldose reductase inhibitor, in diabetic retinopathy. *Arch. Ophthalmol.,* **108,** 1234–1244.

[217] Arauz-Pacheco, C., Ramirez, L. C., Pruneda, L., Sanborn, G. E., Rosenstock, J., and Raskin, P. (1992) The effect of the aldose reductase inhibitor, ponalrestat, on the progression of diabetic retinopathy. *J. Diabetes Complications,* **6,** 131–137.

